# Anti-inflammatory and antinociceptive effects of the isatin derivative (Z)-2-(5-chloro-2-oxoindolin-3-ylidene)-N-phenyl-hydrazinecarbothioamide in mice

**DOI:** 10.1590/1414-431X202010204

**Published:** 2020-09-07

**Authors:** L.L.S.F.R. Dantas, A.G. Fonseca, J.R. Pereira, A.A. Furtado, P.A.T.M. Gomes, M.F. Fernandes-Pedrosa, A.C.L. Leite, M.J.B.M. Rêgo, M.G.R. Pitta, T.M.A.M. Lemos

**Affiliations:** 1 Programa de Pós-Graduação em Desenvolvimento e Inovação Tecnológica em Medicamentos, Universidade Federal do Rio Grande do Norte, Natal, RN, Brasil; 2 Programa de Pós-Graduação em Bioquímica, Universidade Federal do Rio Grande do Norte, Natal, RN, Brasil; 3 Programa de Pós-Graduação em Ciências Farmacêuticas, Universidade Federal de Pernambuco, Recife, PE, Brasil; 4 Departamento de Farmácia, Universidade Federal do Rio Grande do Norte, Natal, RN, Brasil; 5 Departamento de Ciências Farmacêuticas, Universidade Federal de Pernambuco, Recife, PE, Brasil; 6 Núcleo de Pesquisa em Inovação Terapêutica Suely Galdino, Universidade Federal de Pernambuco, Recife, PE, Brasil; 7 Departamento de Análises Clínicas e Toxicológicas, Universidade Federal do Rio Grande do Norte, Natal, RN, Brasil

**Keywords:** Isatin derivative, Thiosemicarbazones, Inflammation, Antinociception, Animal models

## Abstract

Several isatin derivatives have shown important biological activities, which have attracted interest from researchers. For this reason, the present study aimed to evaluate the anti-inflammatory and antinociceptive effects of the isatin derivative (Z)-2-(5-chloro-2-oxoindolin-3-ylidene)-N-phenyl-hydrazinecarbothioamide (COPHCT) in mice. Three doses of this compound were tested: 1.0, 2.5, and 5.0 mg/kg. The anti-inflammatory activity was assessed using the carrageenan-induced paw edema model and the zymosan-induced air pouch model. The evaluation of the antinociceptive effect was performed through the formalin test and the acetic acid-induced abdominal writhing test. The paw edema assay demonstrated that all doses of the compound showed a significant reduction of the edema in the second hour evaluated, but a better response was observed in the fourth hour. The zymosan-induced air pouch model indicated that the compound, in all doses, significantly reduced leukocyte migration and total protein concentration levels. In the formalin test, the doses 1.0, 2.5, and 5.0 mg/kg of COPHCT showed activity only in the second phase, with reduction in paw pain time of 73.61, 79.46, and 73.85%, respectively. The number of abdominal writhings decreased with the increasing dose, but only 5.0 mg/kg COPHCT exhibited a significant response, with a reduction of 24.88%. These results demonstrated the ability of this compound to interfere in the anti-inflammatory activity of edema, vascular permeability, and cell migration. In addition, its possible antinociceptive effect may be related to the dose used.

## Introduction

Among the strategies that may lead to the discovery of new drugs, the identification and use of privileged structures has attracted particular attention. These molecular structures present potent and selective binding properties, as well as the ability to interact with different biological targets through the modification of functional groups (1). In the medical chemistry literature, from 615 structures, 41 substructures were considered to be chemically privileged and only 6, including the indole ring, were considered to be biologically privileged (2).

**Figure 1:**
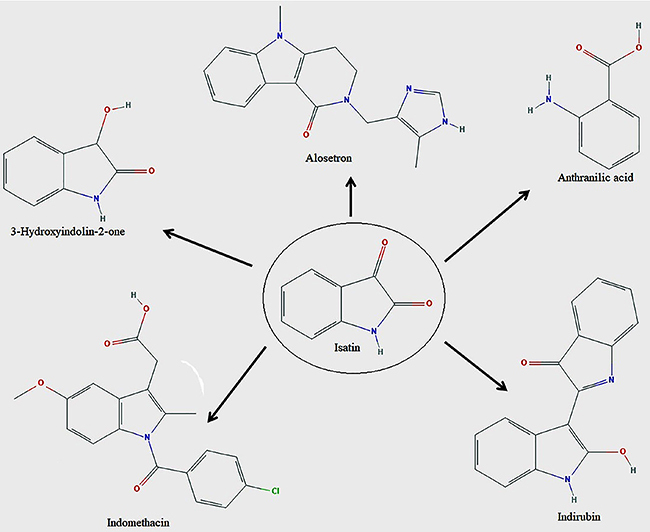
Examples of isatin moiety in drugs.

Isatin (indoline-2,3-dione or indole-1H-2,3-dione) is an indole derivative containing a keto group at position 2 and 3 of the ring. The isatin ring system consists of a pyrrole ring fused with a benzene one (3). The synthetic versatility of isatin has caused a resurgence of interest in the chemistry and bioactivity of its derivatives leading to improvement in procedures of several already known reactions and development of stereoselective methodologies (4). The fascinating application of isatin in organic synthesis is undoubtedly due to the highly reactive C3-keto group (β position), which is extremely susceptible to nucleophilic attack (2). Literature surveys disclose that various isatin derivatives possess pharmacological activities, such as antiviral, antibacterial, anticancer, anticonvulsant, antioxidant, anti-inflammatory, and analgesic (3). Some examples of isatin moiety in drugs are shown in Figure 1.

Inflammation is an attempt to protect the body, removing harmful stimuli and initiating the healing process. On the other hand, pain is a characteristic of inflammation, and therapeutic approaches to pain relief are based on non-steroidal or opioid anti-inflammatory drugs. The inevitable undesirable effects associated with the misuse of these classes of drugs have led to the research and development of newer, safer, and more efficient therapeutic agents, presenting little or no side effect in the control of pain (5,6).

**Figure 2:**
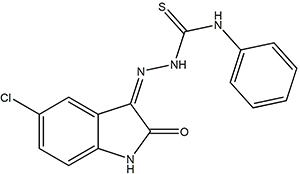
Structure of the isatin derivative (Z)-2-(5-chloro-2-oxoindolin-3-ylidene)-N-phenyl-hydrazinecarbothioamide (COPHCT).

In a previous antitumor screening, the compound LpQM-Int6 (COPHCT as reported herein) (Figure 2) was able to reduce the cell viability of four cancer lines (A.C.L. Leite, P.A.T.M. Gomes, and M.J.B.M. Rêgo, unpublished data). It is well known that inflammation is closely linked to cancer, and many anti-cancer agents are also used to treat inflammatory diseases. Moreover, chronic inflammation increases the risk for various cancers, indicating that elimination of the inflammation may represent a valuable strategy for cancer prevention and therapy (7). In order to investigate other possible activities of an isatin derivative, the objective of this study was to evaluate the effects of the compound COPHCT in nociception and inflammation animal models.

## Material and Methods

### Synthesis of isatin derivative

(Z)-2-(5-chloro-2-oxoindolin-3-ylidene)-N-phenyl-hydrazinecarbothioamide (COPHCT) was obtained from the Laboratory of Planning in Medicinal Chemistry (LpQM) at the Federal University of Pernambuco. This derivative was synthesized from an isatin and a thiosemicarbazide, according to the Karah procedure (8) and it was chemically characterized by nuclear magnetic resonance (NMR), infrared, mass spectrometry, and elemental analyses (A.C.L. Leite and P.A.T.M. Gomes, unpublished data).

### Animals

Male and female Swiss mice (*Mus musculus*), 6-8 weeks of age (25-35 g), were provided by the Animals Laboratory of the Health Sciences Center from the Federal University of Rio Grande do Norte (UFRN). The animals were maintained in a controlled environment (23±2°C, 12 h light/dark cycle, free access to potable water and rodent-specific food). However, they were fasted (for 2 h) of food before the tests. After each assay, the mice were euthanized with an intraperitoneal (*ip*) overdose of thiopental (120 mg/kg) and lidocaine 2% (10 mg/kg) to avoid any suffering. All experimental procedures were performed according to the Brazilian Society of Animal Science and approved by the Committee for Ethics in Animal Experimentation from UFRN (protocol number 088.007-2018).

### Carrageenan-induced paw edema test

This test was adapted from Winter et al. (9). The mice were randomly divided into six groups (n=5): Negative Control (PBS 10.0 mL/kg), Positive Control (PBS 10.0 mL/kg), 2.0 mg/kg dexamethasone (Dex), and COPHCT at 1.0, 2.5, and 5.0 mg/kg. The volume of the right hind paw of the mice was first measured (baseline) using a digital micrometer (Digimess, 100.174BL, Brazil). Then, the animals received a subplantar (*sp*) injection of 50 μL of 1.0% λ-carrageenan (Sigma^®^ Aldrich, USA) or PBS (Negative Control group), and they were treated intragastrically (*ig*), as aforementioned. The percentage of the edema was determined by calculating the difference between the baseline measurement and the right hind paw volume evaluated 0 (soon after treatment administration), 1, 2, 3, and 4 h after treatments (t0, t1, t2, t3, and t4).

### Zymosan-induced air pouch test

The air pouch test was performed according to a previous report (10) with some modifications. Initially, the pouches were produced by a subcutaneous (*sc*) injection of sterile air (5.0 mL) into the dorsal region of the mice. After three days, the pouch was reinforced with additional sterile air (2.5 mL) to maintain the cavity. Six days after the first air injection, the mice were randomly divided into six groups (n=5): Negative Control (PBS 10.0 mL/kg), Positive Control (PBS 10.0 mL/kg), 2.0 mg/kg dexamethasone (Dex), and COPHCT at 1.0, 2.5, and 5.0 mg/kg. Inflammation was induced by a 1.0 mL of zymosan solution (1.0 mg/mL) injection into the preformed pouch (except Negative Control group). Subsequently, each group received the respective treatment (*ig*). After six hours, the animals were euthanized, and the exudates were harvested from the pouches being washed with 2.0 mL of PBS. All samples were centrifuged at 200 *g* for 10 min at 4°C.

The cell pellet was resuspended with 1.0 mL of PBS and diluted in Turk's solution (1:10 v/v). The total leucocyte count was determined using a Neubauer chamber with the aid of a Nikon ECLIPSE E200^®^ (Minato, Japan) microscope at 40× magnification. The data are reported as the number of leukocytes per mL (11).

The supernatants were collected for the determination of total proteins. Each content (10.0 µL) was added to 96-well plates, followed by the addition of 200 µL of Bradford's reagent. Results were obtained using an ELISA microplate reader (BioTek, USA) at 595 nm and reported as µg/mL (12).

### Formalin test

The formalin test was based on the method of Hunskaar and Hole (13). The mice were randomly divided into six groups (n=5): Control (PBS 10.0 mL/kg), 7.5 mg/kg codeine (Cod), 25.0 mg/kg indomethacin (Ind), and COPHCT at 1.0, 2.5, and 5.0 mg/kg. One hour after treatment (*ig*), 2.5% formalin (20 μL) was injected into the ventral surface of the right hind paw of each mouse (*sp* injection). The time (in seconds) the animals spent licking, shaking, and biting the affected paw was rated during two intervals: 0-5 min (phase 1 or neurogenic pain) and 15-30 min (phase 2 or inflammatory pain). The percent reduction of paw pain time was calculated using the formula [(C-T)/C]×100, where C is the paw pain time in the Control group and T is the paw pain time in the treatment group (Cod, Ind, or COPHCT 1.0, 2.5, and 5.0 mg/kg).

### Acetic acid-induced abdominal writhing test

This model was adapted from Mansouri et al. (14). The mice were randomly divided into 5 groups (n=5): Control (PBS 10.0 mL/kg), 25.0 mg/kg indomethacin (Ind), and COPHCT at 1.0, 2.5, and 5.0 mg/kg. Thirty min after treatments (*ig*), abdominal writhes were induced by the *ip* injection of a 0.6% acetic acid solution (10.0 mL/kg). The number of abdominal writhes was counted for 20 min. Results are reported as the total number of abdominal writhes observed per group. The percentage reduction of writhings was calculated using the formula [(C-T)/C]×100, where C is the number of abdominal writhings recorded in the Control group and T is the number of abdominal writhings recorded in the treatment group (Ind or COPHCT at 1.0, 2.5, and 5.0 mg/kg).

### Statistical analysis

The Shapiro-Wilks test was used to determine the distribution of data and Levene's test to evaluate the homogeneity of variance. For a normal distribution, the data are reported as means±SE. Differences between groups were analyzed by one-way analysis of variance (ANOVA) for multiple comparisons of parametric values, followed by the Bonferroni *post hoc* test. In cases in which normality was rejected, Kruskal-Wallis' and Mann-Whitney's U tests were performed. Analyses were performed using the Statistical Package for Social Sciences (SPSS), version 21 (IBM, USA), with a confidence interval of 95%. Differences with P<0.001, P<0.01, and P<0.05 were considered as statistically significant.

## Results

The acute and subchronic toxicity of COPHCT was performed previously, and no toxic effect was shown at the doses used in the assays (1.0, 2.5, and 5.0 mg/kg) (T.M.A.M. Lemos, L.L.S.F.R. Dantas, A.G. Fonseca, and J.R. Pereira, unpublished data).

### Carrageenan-induced paw edema test

**Figure 3:**
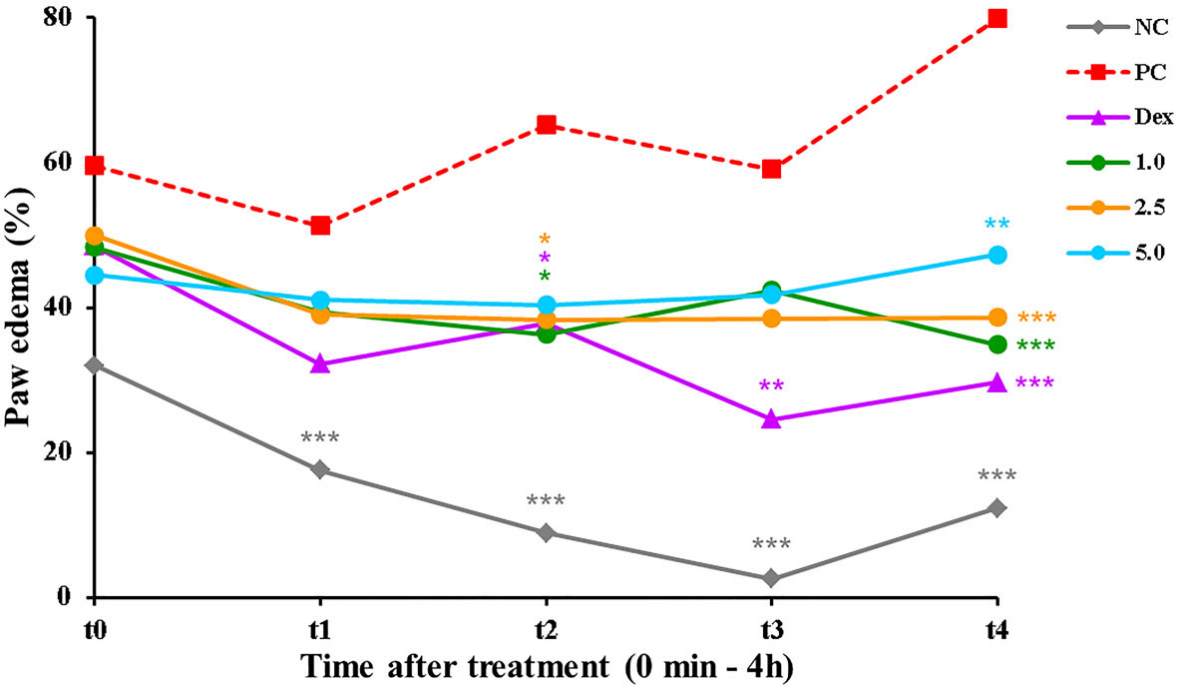
Effect of COPHCT in the paw edema assay induced by the subplantar injection of carrageenan in the hind paw of the mice. Data are reported as means±SE of 5 animals per group. *P<0.05, **P<0.01, and ***P<0.001 compared to the Positive Control group, determined by analysis of variance (ANOVA) followed by Bonferroni post-hoc test. Groups: NC (Negative Control: PBS, *sp* + PBS 10.0 mL/kg, *ig*); PC (Positive Control: carrageenan 1.0%, *sp* + PBS 10.0 mL/kg, *ig*); Dex (carrageenan 1.0%, *sp* + 2.0 mg/kg dexamethasone, *ig*); 1.0, 2.5, and 5.0 (carrageenan 1.0%, *sp* + COPHCT at 1.0, 2.5, and 5.0 mg/kg, *ig*).

In this model, the edema of the Positive Control group was always above 50% and increased over time (t0: 59.55±5.98, t1: 51.27±3.08, t2: 65.19±4.01, t3: 59.08±7.10, t4: 79.88±0.60), which was already expected since the inflammation was treated only with the vehicle. Possibly due to tissue damage caused by the needle, the Negative Control group showed small edema that decreased over time (t0: 32.00±1.73, t1: 17.57±3.40, t2: 8.91±0.56, t3: 2.59±1.37, t4: 12.36±3.08). The Dex group also showed decreased edema over time (t0: 48.45±5.47, t1: 32.24±2.71, t2: 37.83±4.05, t3: 24.59±4.02, t4: 29.70±2.21), with a significant difference compared to the Positive Control group after the second hour (P<0.05). Also in the second hour of evaluation, the COPHCT 1.0 mg/kg (36.33±5.98) and COPHCT 2.5 mg/kg (38.37±7.56) showed a significant decrease (P<0.05) in edema compared to the Positive Control group, but this difference became more significant in the last evaluated time, demonstrating a potential antiedematogenic activity of COPHCT after 4 h of edema measurement: COPHCT 1.0 mg/kg (34.86±5.42, P<0.001); COPHCT 2.5 mg/kg (38.68±7.15, P<0.001); COPHCT 5.0 mg/kg (47.29±5.79, P<0.01) (Figure 3).

### Zymosan-induced air pouch test

**Figure 4:**
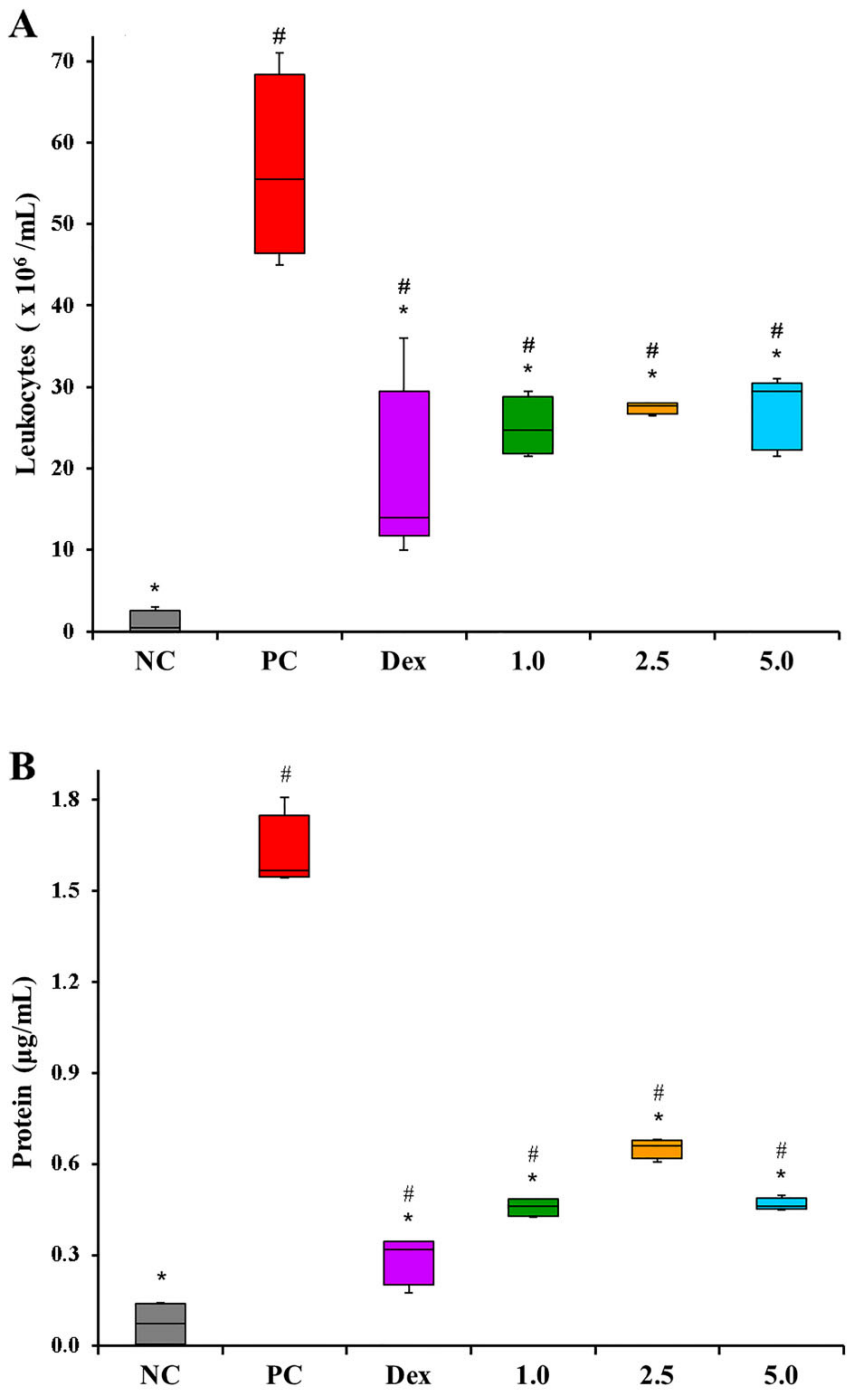
Effect of COPHCT in leukocyte migration (**A**) and extravasation of proteins (**B**) during the air pouch test induced by the subcutaneous injection of 1.0 mg/mL zymosan into the dorsal region of the mice. Central line: median; box: 1st and 3rd quartiles; whiskers: min and max values (5 animals per group). *P<0.05 compared to the Positive Control group and ^#^P<0.05 compared to the Negative Control group, determined by Kruskal Wallis and Mann-Whitney U tests. Groups: NC (Negative Control: PBS, *sc* + PBS 10.0 mL/kg, *ig*); PC (Positive Control: zymosan 1.0 mg/mL, *sc* + PBS 10.0 mL/kg, *ig*); Dex (zymosan 1.0 mg/mL, *sc* + 2.0 mg/kg dexamethasone, *ig*); 1.0, 2.5, and 5.0 (zymosan 1.0 mg/mL, *sc* + COPHCT 1.0, 2.5, and 5.0 mg/kg, *ig*.

This test was used to evaluate other aspects related to inflammatory stimuli. The Positive Control group formed an exudate with a large amount of leukocytes at the site of inflammation, and treatment with COPHCT (all doses) significantly decreased (P<0.05) the inflammatory infiltrate into the air pouch: 55, 50, and 47% for COPHCT at 1.0, 2.5, and 5.0 mg/kg, respectively. Dexamethasone had a similar response, with a reduction of 75% (P<0.05) in leukocyte migration. All the groups evaluated also showed statistically different results (P<0.05) compared to the Negative Control group (Figure 4A).

Similarly, total protein levels were significantly decreased (P<0.05) when comparing the Positive Control group to all other groups: 80% (Dex), 71, 58, and 71% (COPHCT at 1.0, 2.5, and 5.0 mg/kg, respectively). All groups evaluated also showed statistically different results (P<0.05) compared to the Negative Control group (Figure 4B).

### Formalin test

**Figure 5:**
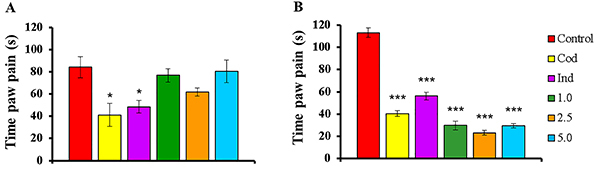
Effect of COPHCT in nociceptive test induced by the subplantar injection of 2.5% formalin in the hind paw of the mice. **A**, Phase 1 or neurogenic pain: 0-5 min. **B**, Phase 2 or inflammatory pain: 15-30 min. Data are reported as means±SE of 5 animals per group. *P<0.05 and ***P<0.001 compared to the Control group, determined by analysis of variance (ANOVA) followed by Bonferroni post-hoc test. Groups: Control (PBS 10.0 mL/kg, *ig*); Cod (7.5 mg/kg codeine, *ig*); Ind (25.0 mg/kg indomethacin, *ig*); 1.0, 2.5, and 5.0: COPHCT 1.0, 2.5, and 5.0 mg/kg, *ig*).

The first phase of the formalin test (0-5 min) indicated that only codeine and indomethacin presented a statistically significant difference (P<0.05) compared to the Control group and they exhibited a reduction in paw pain time of 51.11% (41.16±10.25) and 42.36% (48.53±5.69), respectively (Figure 5A).

In the second phase of the formalin test (15-30 min), all treatments presented results considered significant (P<0.001) compared to the Control group (113.19±4.26), indicating a reduction of paw pain time of 64.17% (Cod: 40.56±2.76), 50.21% (Ind: 56.36±3.45), 73.61% (COPHCT at 1.0 mg/kg: 29.87±3.83), 79.46% (COPHCT 2.5 mg/kg: 23.25±2.10), and 73.85% (COPHCT at 5.0 mg/kg: 29.60±2.02) (Figure 5B). The COPHCT (all doses) showed greater reduction in paw pain time than the standard drugs and showed significant differences mainly with indomethacin (P<0.001). The COPHCT at 2.5 mg/kg was the only one that showed difference with codeine (P<0.05).

### Acetic acid-induced abdominal writhing test

**Figure 6:**
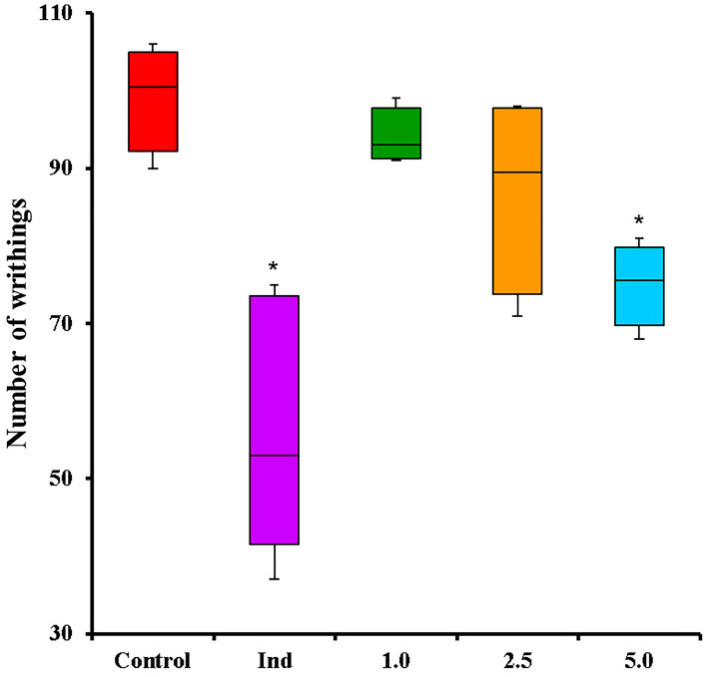
Effect of COPHCT in abdominal writhing test induced by the *ip* injection of 0.6% acetic acid in mice. Central line: median; box: 1st and 3rd quartiles; whiskers: min and max values (5 animals per group). *P<0.05 compared to the Control group, determined by Kruskal Wallis and Mann-Whitney U tests. Groups: Control (PBS 10.0 mL/kg, *ig*); Ind (25.0 mg/kg indomethacin, *ig*); 1.0, 2.5, and 5.0 (COPHCT 1.0, 2.5, and 5.0 mg/kg, *ig*).

The intraperitoneal administration of agents that irritate serous membranes provokes a very typical behavior in mice, which is characterized by abdominal writhing, elongation and extension of the hind limbs, twisting of dorsoabdominal muscles, ataxia, and reduced motor activity (15). In the current report, indomethacin presented a significant reduction (47.26%, P<0.05) in abdominal writhings compared to the Control group. Overall, the number of abdominal writhings decreased with increasing COPHCT doses, but only COPHCT at 5.0 mg/kg presented a significant difference (P<0.05) compared to the Control group, indicating a reduction of 24.88% of abdominal writhings (Figure 6). Furthermore, COPHCT at 5.0 mg/kg was the only dose that showed no significant difference (P>0.05) compared to indomethacin, which indicates that they have similar antinociceptive activity.

## Discussion

According to the literature, thiosemicarbazone derivatives present promising anti-inflammatory activities (16). Thus, in the present research, the anti-inflammatory activity of COPHCT was evaluated using two *in vivo* models: carrageenan-induced paw edema and zymosan-induced air pouch.

The acute inflammatory response is characterized by increased vascular permeability and cellular infiltration leading to edema formation, as a result of fluid and protein extravasation and leukocyte accumulation at the site of inflammation (17). The carrageenan-induced paw edema model is the simplest and most widely used method to study anti-inflammatory activity, especially the antiedematogenic activity of chemical compounds (18). Carrageenan, as the phlogistic agent, induces an acute inflammatory process associated to hyperalgesia and characterized by vasodilation due to the release of inflammatory mediators, which is characteristic of an edematogenic response (19).

Following the evaluation of the anti-inflammatory activity at the level of edema, COPHCT was tested by the air-pouch model, using zymosan as an inflammatory agent, to assess its activity on cell migration processes and extravasation of proteins. Zymosan can activate the complement alternative pathway and induce the production of proinflammatory mediators such as C5 peptide, which is a potent neutrophil chemoattractant (20). In addition, a cascade of events that culminates in the inflammatory process is triggered when zymosan particles are internalized by phagocytic cells after contact with cell surface receptors, such as Dectin-1 and Toll-like receptor 2 (TRL-2) (21).

Evaluating the results of paw edema and air pouch tests, we can see that all doses of COPHCT showed an inhibitory action from the most classic signs of inflammation, such as edema formation, as well as in cellular activation processes, generally more harmful, reducing the migration of leukocyte cells (mainly neutrophils that are more present in the acute inflammatory process). Dexamethasone, the standard drug used in these models, is a synthetic glucocorticoid with anti-inflammatory and immunosuppressant properties (22). Therefore, it is possible to suggest that COPHCT (1.0, 2.5, and 5.0 mg/kg) showed a similar response to this drug because there was no statistical difference between them, as well as between the doses of COPHCT.

It has been shown that isatin derivatives, which possess electron withdrawing groups such as halogens, at the C5 or C7 position exhibit remarkable anti-inflammatory activity (23,24). Thus, it is also possible to suggest that the anti-inflammatory activity of COPHCT may be attributed to the presence of the chlorine in C5 position in the structure.

Furthermore, recent investigations revealed the antinociceptive property of several isatin derivatives (5,25,26). Therefore, this study has also investigated the antinociceptive activity of COPHCT through two experimental models.

The formalin-induced test is considered the most used model for screening new compounds with antinociceptive activity because it is the one that most resembles clinical pain compared to other tests (27). This nociception test has two pain phases and it involves different mechanisms. The first phase of pain (or neurogenic pain) is attributed to the direct activation of nociceptors and afferent primary fibers that release bradykinin and substance P (13). It is believed that the second phase (or inflammatory pain) is mediated by peripheral and medullary sensitization, causing release of inflammatory mediators such as histamine, prostaglandins, serotonin, and bradykinin (28).

Indomethacin is a non-steroidal anti-inflammatory indole acetic acid derivative that inhibits the activity of the enzyme cyclooxygenase (COX), the enzyme responsible for catalyzing the rate-limiting step in prostaglandin and thromboxane synthesis via the arachidonic acid pathway (15). It is a drug with potent antipyretic, analgesic, and anti-inflammatory activities that has been effectively used in the management of mild-to-moderate pain (29). On the other hand, codeine is an opiate agonist in the central nervous system commonly used for chronic pain states. It acts primarily on Mu opioid receptors, but first it needs to be metabolized to morphine by the body for it to display any activity (30). In this study, the results indicated that all doses of COPHCT exhibited a better antinociceptive response than indomethacin in the inflammatory phase. Drugs that act on the first phase of the formalin test present a central action as narcotics. When the reduction of pain occurs in the second phase, it suggests a peripheral action similar to the non-steroidal anti-inflammatory (NSAID) drugs and anti-inflammatory steroids (15). Only good responses in the second phase of the formalin test are a typical features of COX inhibitors (31).

These antinociception results corroborate the data obtained in the evaluation of the anti-inflammatory activity presented by COPHCT, since this compound seems to act in the inflammatory phase of pain. However, another study with thiosemicarbazone revealed activity in both phases of inflammation induced by carrageenan, suggesting an action in the early mediators of inflammation or through inhibition of pro-inflammatory cytokines (6).

The acetic acid-induced abdominal writhing assay has been used to assess antinociceptive properties of many compounds with various mechanisms of action. It is a sensitive method for evaluating peripherally acting analgesics on inflammatory and visceral pain. The pain sensation in acetic acid-induced writhing is due to local inflammatory response resulting from the release of endogenous mediators that stimulate the peripheral nociceptive neurons. In mice, it promotes an increase in the levels of prostaglandins (PGE2 and PGF2α) and lipoxygenase products as well as serotonin and histamine and the release of bradykinin and cytokines (tumor necrosis factor-α and interleukin-1β) (15,27,32).

Researchers investigated the biological activities of derivatives of indole and isatin oximes and observed that compounds that possess a chlorine in the C5 position exhibited high analgesic and anti-inflammatory activities (33). Furthermore, other studies reported that unsubstituted isatins are 5-10 times less active than halogenated ones at C5 position (34,35). The incorporation of halogen atoms improves membrane permeability and oral absorption, metabolic and chemical stability, or potency, because the formation of halogen bonds may increase protein-ligand stability and contribute to the affinity of drug-receptor binding (36).

In addition, new isatin derivatives were synthesized by reacting thiosemicarbazides with formaldehyde and various secondary amines. After showing antinociceptive and anti-inflammatory activities in animal models, it was concluded that the good response may be due to their structural similarity to indomethacin (37).

In conclusion, the isatin-thiosemicarbazone derivative COPHCT exhibited good anti-inflammatory and antinociceptive activities, mainly in response to signs of inflammation, but further studies are needed to better investigate its pharmacological actions.
